# Comprehensive Metabolite Profiling of Cinnamon (*Cinnamomum zeylanicum*) Leaf Oil Using LC-HR/MS, GC/MS, and GC-FID: Determination of Antiglaucoma, Antioxidant, Anticholinergic, and Antidiabetic Profiles

**DOI:** 10.3390/life13010136

**Published:** 2023-01-03

**Authors:** Muzaffer Mutlu, Zeynebe Bingol, Eda Mehtap Uc, Ekrem Köksal, Ahmet C. Goren, Saleh H. Alwasel, İlhami Gulcin

**Affiliations:** 1 Vocational School of Applied Sciences, Gelişim University, Istanbul 34315, Turkey; 2 Department of Medical Services and Techniques, Tokat Vocational School of Health Services, Gaziosmanpasa University, Tokat 60250, Turkey; 3 Department of Chemistry, Faculty of Science, Ataturk University, Erzurum 25240, Turkey; 4 Department of Chemistry, Faculty of Science and Arts, Erzincan Binali Yildirim University, Erzincan 24100, Turkey; 5 Department Chemistry, Faculty of Sciences, Gebze Technical University, Kocaeli 41400, Turkey; 6 Department of Zoology, College of Science, King Saud University, Riyadh 11362, Saudi Arabia

**Keywords:** *Cinnamomum zeylanicum*, polyphenol, cinnamon oil, antioxidant activity, LC-HRMS, GCMS, enzyme inhibition

## Abstract

In this study, for the first time, the antioxidant and antidiabetic properties of the essential oil from cinnamon (*Cinnamomum zeylanicum*) leaves were evaluated and investigated using various bioanalytical methods. In addition, the inhibitory effects of cinnamon oil on carbonic anhydrase II (hCA II), acetylcholinesterase (AChE), and α-amylase, which are associated with various metabolic diseases, were determined. Further, the phenolic contents of the essential oil were determined using LC-HRMS chromatography. Twenty-seven phenolic molecules were detected in cinnamon oil. Moreover, the amount and chemical profile of the essential oils present in cinnamon oil was determined using GC/MS and GC-FID analyses. (*E*)-cinnamaldehyde (72.98%), benzyl benzoate (4.01%), and *trans*-Cinnamyl acetate (3.36%) were the most common essential oils in cinnamon leaf oil. The radical scavenging activities of cinnamon oil were investigated using 1,1-diphenyl-2-picryl-hydrazil (DPPH^•^), 2,2′-azino-bis(3-ethylbenzthiazoline-6-sulfonic acid), and (ABTS^•+^) bioanalytical scavenging methods, which revealed its strong radical scavenging abilities (DPPH^•^, IC_50_: 4.78 μg/mL; and ABTS^•+^, IC_50_: 5.21 μg/mL). Similarly, the reducing capacities for iron (Fe^3+^), copper (Cu^2+^), and Fe^3+^-2,4,6-tri(2-pyridyl)-S-triazine (TPTZ) were investigated. Cinnamon oil also exhibited highly effective inhibition against hCA II (IC_50_: 243.24 μg/mL), AChE (IC_50_: 16.03 μg/mL), and α-amylase (IC_50_: 7.54μg/mL). This multidisciplinary study will be useful and pave the way for further studies for the determination of antioxidant properties and enzyme inhibition profiles of medically and industrially important plants and their oils.

## 1. Introduction

Plants and their biologically active compounds are among the leading potential resources for the development of new products for different industrial applications [1]. At present, several biologically active compounds derived from plants are being used effectively in various applications, especially in pharmacology [2]. Plants act as living chemical factories that produce several secondary metabolites and are beneficial to the environment. Medicinal plants are of crucial importance, especially in the health, cosmetic, food, and pharmaceutical industries. Turkey has a rich plant biodiversity that can be exploited for use in these areas [3,4]. The main reasons for the use of secondary metabolites in folk medicine are that they are harmless and biologically active. The notable properties of these metabolites include antidiabetic, anti-inflammatory, antioxidant, and neuroprotective effects [5,6].

Naturally occurring antioxidants derived from several plants can act as reactive oxygen species (ROS) or free-radical scavengers or metal chelators in various biological systems [7,8]. Excessive formation of ROS and free radicals in living organisms can induce various chronic and degenerative diseases, including age-related pathologies, cardiovascular disorders, cancer, immunodeficiency syndrome, arteriosclerosis, obesity, and diabetes, by acting on several biomolecules, such as carbohydrates, proteins, lipids, and nucleic acids [9,10]. The presence of antioxidants in biological systems, even at low concentrations, is important to eliminate the adverse effects of ROS and free radicals [11,12]. The biological activities of phenolic compounds obtained from plant sources, particularly their antioxidant properties, have been intensively investigated [13]. Plants exhibit extensive and excellent biological activities [14]. Owing to their biological activities, all living systems, including plants, can remain healthy. In addition, plants contain biologically active regulators and, thus, have a significant therapeutic effect on some chronic diseases [15]. Plant-derived phenolics are in great demand and are being used worldwide because they are less toxic. Therefore, there is a great incentive and demand for plants to treat different diseases. Several herbal medicines that are widely used in the treatment of Alzheimer’s disease (AD) are also known [16].

The genus *Cinnamomum* belongs to the fairly large family Lauraceae, comprising more than 30 genera and approximately 2000–2500 species. *Cinnamomum* species are used as natural antioxidant sources because of their high phenolic content due to the presence of proanthocyanidins and flavonoids. Species of the genus *Cinnamomum* include *C. cassia*, *C. zeylanicum* (*C. verum*), *C. loureiroi,* and *C. burmannii*, which are the most-known ones [17]. However, *C. zeylanicum* is the most studied species. Dried aromatic peel is the main commercial product of *C. zeylanicum* [18], with various therapeutic effects, including antifungal activity. It was reported that that *C. zeylanicum* oil is safe for human consumption. The essential oil of *C. zeylanicum* has antibacterial effects that can combat drug-resistant and ulcer-causing *H. pylori.* *C. zeylanicum* has long been used as a health-promoting agent [19]. Additionally, its essential oil has a protective effect against gastroenteritis [20]. Essential oils from the bark of *C. zeylanicum* are used for their antioxidant, anti-Alzheimer’s disease, anti-skin-whitening, and antidiabetic activities. The leaves and bark of *C. zeylanicum* are commercially valuable owing to their high contents of (*E*)-cinnamylacetate and eugenol (*E*)-cinnamaldehyde. In addition, (*E*)-cinnamaldehyde, the major compound found in the bark oil from *C. zeylanicum*, has various bioactivities [21]. The root of *C. zeylanicum* contains camphor as the main compound.

AD is a neurodegenerative disease that causes cognitive impairment, behavioral abnormalities, and dementia in elderly individuals [22]. Although complete cure of AD is not currently possible, its clinical course can be altered or slowed. Patients with AD generally exhibit three characteristic pathological symptoms, including dementia due to hyper phosphorylation of tau, low acetylcholine (ACh) levels, and amyloid-β aggregation, causing loss of neuronal functions [23]. Of these, the cholinergic hypothesis involving low ACh levels is a clinically viable medical strategy. Thus, strategies to inhibit cholinergic acetylcholinesterase (AChE) in brain tissue have gained importance [24,25]. AChE, a hydrolytic enzyme, converts ACh to acetate and choline (Ch), whereas butyrylcholinesterase (BChE) converts butyrylcholine (BCh) to butyrate and Ch [26]. AD is clinically affected by brain tissue damage caused by ACh deficiency [27]. In addition, many factors, such as environmental conditions and genetic predisposition, greatly affect the variability in the amount of ACh [28]. Particularly, AChE inhibitors of natural origin increase cholinergic transmission, exerting therapeutic effects on patients with AD [29,30,31].

The use of plant and plant-derived components also affects biological and pathological events related to diabetes complications [32]. Metformin, a plant-derived compound, is widely used to decrease blood sugar levels and indirectly reduce diabetes mellitus (DM), a global health problem provoked by hyperglycemia and characterized by the absence of or resistance to insulin [33]. As a result of this chronic metabolic disease, unusual sugar levels in blood plasma cause neuropathy, cardiovascular disorders, retinopathy, and nephropathy [34]. Hydrolysis of dietary polysaccharides into glucose leads to an increase in postprandial glucose levels [35]. Patients with diabetes have adversity in blood sugar transport to cells after digestion. In this case, the blood sugar level remains high while the cells are starved [36]. Additionally, α-amylase and α-glucosidase enzymes hydrolyze polysaccharides into monosaccharides for absorption from the small intestine. Therefore, their inhibition is of great importance in the treatment of type-2 diabetes mellitus (T2DM) [37,38]. Thus, digestive enzyme inhibitors (DEIs) obtained from natural sources can be used for the treatment of hyperglycemia and T2DM [39]. DEIs act as antidiabetic drugs and decrease blood sugar levels by reducing intestinal carbohydrate absorption. Thus, DEIs compete with oligosaccharides for binding to the active site of the enzyme. Acarbose is one of the best examples of this form of inhibition [40].

Carbonic anhydrases (CAs) catalyze the reversible hydration and dehydration of carbon dioxide (CO_2_) to protons and bicarbonate (HCO_3_^−^) [41]. Since the approval of dorzolamide in 1995, CA isoenzyme inhibitors (CAIs) have been widely used to treat glaucoma [42]. Five years later, in 2000, a second clinical CA inhibitor, brinzolamide, was used in the treatment of glaucoma in many European countries. Glaucoma causes irreversible peripheral vision loss and, consequently, high intraocular pressure (IOP), causing blindness [43]. Glaucoma, an eye disease that damages the optic nerve and causes vision loss, is one of the leading causes of blindness worldwide. It is also estimated that the number of people with glaucoma will exceed 115 million worldwide by 2040 [44,45]. Although laser, surgical, and pharmacological treatments are available for glaucoma, the clinical application of CA II isoenzyme inhibitors in epithelial cells in the ciliary body reduces aqueous humor secretion and consequently reduces IOP [46]. CA II inhibitors, including methazolamide, acetazolamide, dichlorphenamide, and ethoxzolamide, are pressure-lowering systemic drugs for glaucoma treatment [47]. However, these inhibitors inhibit the localization of CA isoforms other than those in the eye, causing undesirable side effects, including paresthesia, fatigue, and serious concerns [48,49]. To avoid these undesirable side effects, inhibitors with topical activity and natural origin are preferred. Therefore, we present cinnamon leaf oil for the treatment of glaucoma to the attention of researchers working on this subject.

In our current study, we evaluated the chemical composition and antioxidant, anti-Alzheimer’s disease, and antidiabetic effects of cinnamon leaf oil. We also identified the polyphenol and essential oil contents of cinnamon leaf oil using LC-HRMS and GS-MS/FID.

## 2. Materials and Methods

### 2.1. Chemicals

2,2′-azino-bis(3-ethylbenzthiazoline-6-sulfonic acid) (ABTS), α-tocopherol, 2,9-dimethyl-1,10-phenanthroline (neocuproine), Trolox, butylated hydroxyanisole (BHA), 1,1-diphenyl-2-picryl-hydrazyl (DPPH^•^), and butylated hydroxytoluene (BHT) were purchased from Sigma-Aldrich Chemie GmbH (Steinheim, Germany). Ascorbic acid, fumaric acid, chlorogenic acid, caffeic acid, naringin, vanillic acid, syringic acid, rutin, rosmarinic acid, p-coumaric acid, salicylic acid, quercetin, luteolin, naringenin, chrysin, and emodin were purchased from Sigma-Aldrich. Hyperoside, luteolin 7-glycoside, orientin, (+)-trans-taxifolin, quercitrin, hispidulin, apigenin, hederagenin, and acacetin were obtained from TRC, Canada. Verbascoside and luteolin-7-rutinoside were purchased from HWI Analytik GMBH and Carbosynth Ltd., respectively. Hesperidin was purchased from J&K Co., Ltd. Isosakuranetin, dihydrokaempferol, and penduletin were purchased from Phytolab. Apigenin 7-glucoside was obtained from the EDQM CS. Myricetin was purchased from Carl Roth GmbH & Co. Nepetin and caffeic acid phenethylester were purchased from Supelco and the European Pharmacopoeia, respectively.

### 2.2. Preparation of Cinnamon (C. zeylanicum) Leaf Oil

Cinnamon (*C. zeylanicum*) leaves were obtained from a local market. Cinnamon leaf oil was prepared using the steam distillation method. This method is a multistage continuous distillation process using steam as the stripping gas to obtain the oils. Steam is applied directly to the plant. The vapor mixture is collected and condensed to obtain a liquid in which water and oil form two different layers. The upper part of these layers is essential oil containing hydrophobic compounds, and the lower part is a hydrolysate or hydrosol containing hydrophilic components. Polar compounds remaining in the water can be recovered by the cohobating process. Dried cinnamon leaves were placed on a grid above the steam inlet of the steam distillation unit. Steam was supplied to the unit for approximately 2 h. The mixture containing water vapor and volatilized essential oils was condensed using a cooler and collected in the collection container. The cinnamon leaf oil accumulated in the collection container formed a separate layer from that of water owing to the density difference and was extracted.

### 2.3. Polyphenolic Composition Using LC-HRMS Analysis

LC-HRMS experiments were carried out using a Thermo Orbitrap Q-Exactive mass spectrometer (Thermo Fisher Scientific Inc., Waltham, MA, USA), equipped with a Troyasil C18 column (150 × 3 mm i.d., 3 µm particle size) (Istanbul, Turkey). The mobile phases A and B were composed of 1% formic acid–water and 1% formic acid–methanol, respectively. The gradient programs were 0–1.00 min, 50% A and 50% B; 1.01–6.00 min, 100% B; and finally, 6.01–10 min, 50% A and 50% B [29]. The flow rate of the mobile phase was 0.35 mL/min, and the column temperature was set to 22 °C. Environmental conditions were set as 22.0 ± 5.0 °C temperature and 50 ± 15% relative humidity [50]. According to our previous experiences and reported data from the literature, an acidified methanol and water gradient was determined to be the best solvent system to obtain suitable ionization abundance and separation of compounds in HPLC. Because the electrospray ionization (ESI) source provides one of the best ionizations for small and relatively polar compounds, we chose the ESI source for the applied method. The ions between *m/z* 85 and 1500 were scanned in the high-resolution mode of the instrument [50]. Compounds were identified by comparing their retention time and HRMS data with those of standard compounds (in the range of purity 95–99%; see Section 2.1). Dihydrocapsaicin (purity 95%) was used as an internal standard for LC-HRMS measurements to reduce the repeatability problem caused by external effects, such as ionization repeatability, in mass spectrometry measurements. The mass parameters of each target compound are listed in Table 1. Further details of the LC-HRMS method, uncertainty evaluation methodology, and confirmation parameters for phenolics were provided in detail previously [50,51,52].

Validation of the LC-HRMS method was carried out using analytical standards of target compounds using negative or positive ions, listed in Table 1. Dihydrocapsaicin was used as the internal standard. Method validation parameters were selectivity, linearity, recovery, repeatability, intermediate precision, limit of detection (LOD), and limit of quantification (LOQ). The LODs of the method for individual compounds were determined using the following equation: LOD or LOQ = κSD*a*/*b*, where LOQ is 3 and κ = 3 for LOD. Also, SD*a* represents the standard deviation of the intercept, and *b* represents the slope. The detailed validation procedure and uncertainty assessment methodology of the applied method was reported in our previous paper [49,50,51,52].

### 2.4. Essential Oil Isolation and GC/MS and GC-FID Analyses of Cinnamon Leaf Oil

The extract was dried over anhydrous CaCl_2_, and the essential oil was stored at 4 °C until the GC-MS/FID measurements. The yield of oil was 1.52%. The GC-MS analysis was carried out using a Thermo Scientific Trace GC 1310 connected to a Thermo TSQ 9610 MS system on a DB-5 capillary column (60 m × 0.25 mm, 0.25 mm film thickness) with helium as the carrier gas (0.8 mL/min). GC oven temperature was maintained at 80 °C for 10 min, programmed to 280 °C at a rate of 4 °C/min, and kept constant at 280 °C for 5 min. The split ratio was adjusted to 1:20. The injector temperature was set to 250 °C. The mass spectra were recorded at 70 eV. The mass range was *m/z* 35–650. GC-FID analysis was performed using a Thermo Scientific Trace GC 1310 instrument. The FID detector temperature was 280 °C. To obtain the same elution order as GC-MS, simultaneous auto-injection was performed in duplicate on the same column under the same operational conditions. The relative percentage of the separated compounds was calculated from the FID chromatograms [53,54,55]. Alkanes were used as reference points in the calculation of Kovats Indices (KI). Compounds were identified by comparing their retention times and mass spectra with those obtained from authentic samples and/or the NIST and Wiley spectra, as well as literature data [54,55,56,57].

### 2.5. Reducing Ability Assays

The Fe^3+^ reduction potential of cinnamon leaf oil was determined using the Fe^3+^(CN^−^)_6_ complex reduction method [58]. For this purpose, various concentrations of cinnamon leaf oil were transferred to test tubes, and 2.5 mL of phosphate buffer (pH 6.6, 0.2 M) and 2.5 mL of [K_3_Fe(CN)_6_] solutions (1%) were added. Then, the mixture was vortexed and incubated at 50°C for 25 min. A portion of trichloroacetic acid (10%, 2.5 mL) was added. Then, 2.5 mL of upper layers of the solutions was mixed with 2.5 mL distilled water and 0.5 mL FeCl_3_ (0.1%). The absorbance of reducing effects of cinnamon leaf oil was spectrophotometrically recorded at 700 nm.

After all the necessary experimental procedures, the absorbance of Cu^2+^ reducing ability of cinnamon leaf oil was determined according to a prior study [59]. For this purpose, 0.25 mL CuCl_2_ solution (10 mM), 0.25 mL ethanolic neocuproine solution (7.5 × 10^−3^ M), and 250 μL NH_4_Ac buffer solution (1.0 M) and different concentrations (10–30 μg/mL) of test tubes containing cinnamon leaf oil were transferred. Then, the total volume was increased to 2 mL with distilled water and their absorbance values were recorded at 450 nm after 30 min of incubation.

Lastly, the Fe^3+^-TPTZ complex reducing ability reduction ability cinnamon leaf oil was performed according to a previous study [60]. For this, 2.25 mL of TPTZ solution (10 mM in 40 mM HCl) was freshly prepared and transferred to 2.5 mL of acetate buffer (0.3 M, pH 3.6) and 2.25 mL of FeCl_3_ solution (20 mM). Then, different concentrations of cinnamon leaf oil were transferred and incubated at 37 °C for 25 min. Finally, the absorbance of reducing power of cinnamon leaf oil was spectrophotometrically measured at 593 nm. All experiments related to reducing abilities were repeated three times and the results were given as the arithmetic mean of these repetitions.

### 2.6. Radical Scavenging Activities

The radical scavenging potential of cinnamon leaf oil was evaluated using the DPPH radical according to the Blois method [61]. Briefly, 1 mL of DPPH^•^ solution (0.1 mM), which was prepared in ethanol and possessed a blue color, was added to the cinnamon leaf oil at different concentrations (10–30 μg/mL). Then, they were incubated at room temperature for 25 min and their absorbance values were recorded at 517 nm. ABTS radical scavenging ability of cinnamon leaf oil was realized according to Gulcin’s method [62]. Firstly, aqueous solution of ABTS (7.0 mM) was oxidized by oxidants such as K_2_S_2_O_8_ (2.5 mM) for generation of its radical cation (ABTS^•+^). The ABTS^•+^ solution was diluted with a phosphate buffer (0.1 M, pH 7.4) prior to use, adjusting the absorbance value of the control to 0.750 ± 0.025 at 734 nm. Then, 1 mL of ABTS^•+^ solution was added to 3 mL of cinnamon leaf oil at different concentrations (10–30 μg/mL). After 30 min, the remaining absorbance of ABTS^•+^ was measured at 734 nm [63].

The radical scavenging potential (RSC) of cinnamon leaf oil was calculated using the formula: RSC (%) = (1 − A_c_/A_s_) × 100, where A_c_ and A_s_ are the absorbance values of the control and sample, respectively. In addition, IC_50_ was obtained from the graphs as µg/mL [64].

### 2.7. Acetylcholinesterase Inhibition Assay

The inhibitory effects of cinnamon leaf oil on AChE from horse serum and *Electrophorus electricus* were evaluated according to the methods described in our previous studies [65]. Acetylthiocholine iodide (AChI) and 5,5′-dithio-bis-(2-nitrobenzoic acid) (DTNB) were used as substrates [66]. Briefly, 1 mL of Tris/HCl buffer (1.0 M, pH 8.0), 10 μL of different concentrations of cinnamon leaf oil, and 50 μL AChE were mixed in a test tube. Then, the sample was incubated at 25 °C for 15 min and 50 μL of DTNB solution (0.5 mM,) was transferred. Then, the reaction was started by adding 50 μL of AChI solution (10 mM), and absorbance was recorded at 412 nm [67].

### 2.8. α-Amylase Inhibition Assay

The inhibitory potential of cinnamon leaf oil on α-amylase was determined using a starch substrate according to the Xiao procedure [68]. First, 1 g starch was dissolved in 50 mL NaOH solution (0.4 M) and heated at 80 °C for 20 min. After cooling, the pH was adjusted to 6.9, and the volume was adjusted to 100 mL using distilled water. Next, 35 µL of starch solution, 35 µL of phosphate buffer (pH 6.9), and 5 µL of the cinnamon leaf oil solutions were mixed. After incubation at 37 °C for 20 min, 20 µL of α-amylase solution was added and incubated again for 20 min. The reaction was completed by adding 50 µL of 0.1 M HCl, and absorbance was measured at 580 nm [69].

### 2.9. hCA II Inhibition Assay

Human erythrocytes were used as a source of CA II [70]. CA II was purified to high purity using the Sepharose-4B-tyrosine-sulfanilamide affinity column technique [71,72]. The protein content at each step of the purification assay was determined according to the Bradford method [73], and bovine serum albumin was used as the standard protein [74]. The purity of hCA II was determined using SDS-PAGE, as described in our previous work [75]. During hCA II purification and inhibition studies, esterase activity assay was measured spectrophotometrically at 348 nm [76].

### 2.10. Determination of IC_50_ Value

IC_50_ values were calculated to determine the potency of the inhibitory effects of cinnamon leaf oil. IC_50_ values were calculated from the graphs derived from the enzyme activity corresponding to increasing amounts of cinnamon leaf oil [77].

### 2.11. Statistical Analysis

Statistical analyses were performed using Student’s *t*-test (GraphPad Prism 6, GraphPad, La Jolla, CA, USA, Software 7.0). The data are presented as means ± standard deviations (SD). The minimum significance level was set at *p* < 0.05.

## 3. Results

### 3.1. Polyphenolic Composition of Cinnamon Leaf Oil

The LC-HRMS assay was validated by determining the linearity, selectivity, precision, recovery, matrix effect, accuracy, and stability of the analytes [78,79,80]. In this study, 28 phenolic compounds were tentatively identified from cinnamon leaf oil. The LC-HRMS results showed that cinnamon leaf oil is rich in hispidulin (9.98 mg/L oil), herniarin (7.82 mg/L oil), and apigenin (6.61 mg/L oil). The cinnamon leaf oil was obtained by liquid-liquid extraction in order to determine the secondary metabolite profile of the species. A total of 100 mg of cinnamon leaf oil was dissolved by mobile phase B (1% formic acid in methanol) in a 4 mL volumetric flask and kept in an ultrasonic bath for 10 min. Then, 100 μL of internal standard (dihydrocapsaicin solution in methanol) was adjusted to volume with mobile phase B. The final solution was filtered through a 0.45 μm Millipore Millex-HV filter and the final solution (1 mL) was transferred into a capped auto sampler vial, from which 2 μL of sample was injected into LC for each run. The samples in the auto sampler were kept at 15 °C during the experiment [81].

**Figure 1:**
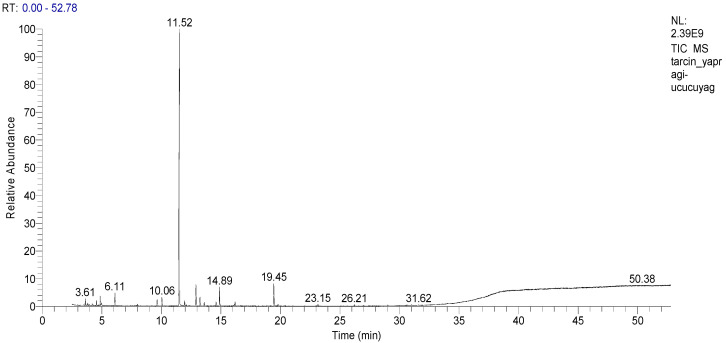
Compounds and their % ratios determined using GCMS analysis of the oil sample obtained from cinnamon (*C. zeylanicum*) leaf.

GC-MS was used to perform accurate qualitative analysis of the contents of different aromatic compounds and essential oils [80]. Table 2 presents the relative information on the aromatic components of cinnamon leaf oil. In the present study, 17 volatile components were identified in cinnamon leaf oil samples. Among these, *E*-cinnamaldehyde (72.98%), benzyl benzoate (4.01%), β-caryophyllene (3.45%), and *trans*-cinnamylacetate (3.36%) were the most abundant compounds in cinnamon leaf oil (Figure 1 and Table 2).

### 3.2. Reducing Ability of Cinnamon Leaf Oil

**Figure 2:**
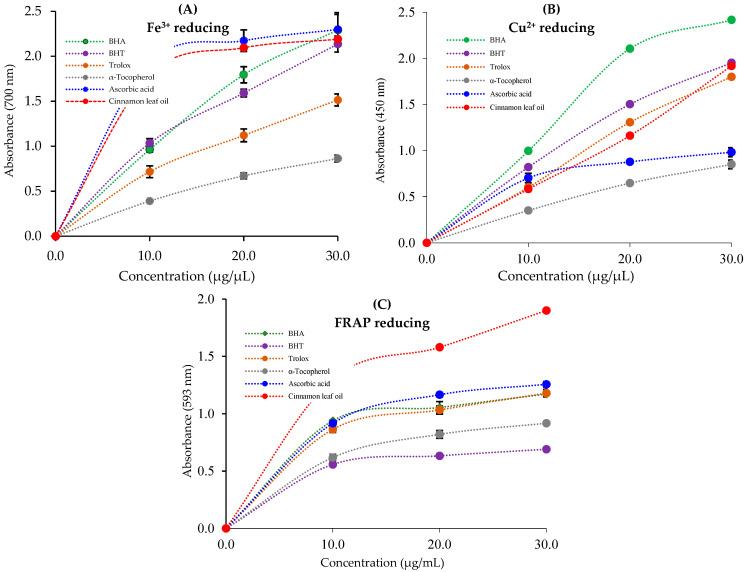
Fe^3+^, Cu^2+^, and Fe^3+^-TPTZ reducing ability of cinnamon (*C. zeylanicum*) leaf oil and standards.

Cinnamon (*C. zeylanicum*) leaf oil demonstrated strong and effective reducing capacity in Fe[Fe(CN^−^)_6_]_3_, Fe^3+^-TPTZ, and Cu^2+^ reduction assays [82]. First, an Fe^3+^–Fe^2+^ conversion assay was performed to measure the reducing ability of cinnamon leaf oil (Figure 2A and Table 3). Cinnamon leaf oil and standards at a concentration of 50 µg/mL (r^2^ = 0.9804) exhibited Fe^3+^ reducing ability (*p* < 0.01) in the following order: ascorbic acid (2.298±0.086, r^2^ = 0.9659) ≥ BHA (2.292 ± 0.012, r^2^ = 0.9993) ≥ cinnamon leaf oil (2.190 ± 0.039, r^2^ = 0.9741) ≥ BHT (2.136 ± 0.090, r^2^ = 0.9957) > Trolox (1.514 ± 0.066, r^2^ = 0.9963) > α-tocopherol (0.862 ± 0.038, r^2^ = 0.9996). The increased absorbance reflects the complex formation and an increased reducing ability (Figure 1A).

In addition to the Fe^3+^-reducing effect, the Fe^3+^-TPTZ and Cu^2+^-reducing abilities of cinnamon leaf oil were investigated and are summarized in Figure 2B, C and Table 3 (r^2^ = 0.9783). Cinnamon leaf oil showed high absorbance values at the tested concentrations. Cinnamon leaf oil and standards at a concentration of 30 μg/mL reduced Cu^2+^ ions in the following order (Figure 2B): BHA (2.292±0.012, r^2^ = 0.9993) > BHT (1.953 ± 0.045, r^2^ = 0.9998) > cinnamon leaf oil (1.918 ± 0.031, r^2^ = 0.9992) > Trolox (1.800 ± 0.096, r^2^ = 0.9974) > ascorbic acid (0.983 ± 0.048, r^2^ = 0.9822) > α-tocopherol (0.851 ± 0.046, r^2^ = 0.9994).

Cinnamon (*C. zeylanicum*) leaf oil had an effective reducing potential in this reduction assay (Table 3 and Figure 2C). Test materials containing cinnamon leaf oil and standards declined in the following order of FRAP reducing capacity: cinnamon leaf oil (1.900 ± 0.021, r^2^ = 0.9725) > ascorbic acid (1.257 ± 0.024, r^2^ = 0.9869) > Trolox (1.180 ± 0.032, r^2^ = 0.9732) ≥ BHA (1.172 ± 0.014, r^2^ = 0.9605) > α-tocopherol (0.918 ± 0.011, r^2^ = 0.9904) > BHT (0.690 ± 0.008, r^2^ = 0.9645).

### 3.3. Radicals Scavenging Effect of Cinnamon Leaf Oil

**Figure 3:**
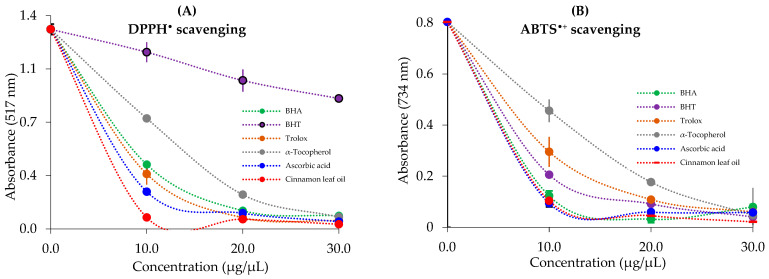
1,1-diphenyl-2-picryl-hydrazyl free radical (DPPH^•^) scavenging and 2,2′-azino-bis(3-ethylbenzthiazoline-6-sulfonic acid (ABTS^•+^) scavenging effects of cinnamon (*C. zeylanicum*) leaf oil and standards.

The IC_50_ values of the DPPH scavenging effects of cinnamon (*C. zeylanicum*) leaf oil and standard radical scavengers were in the following order: 4.78 µg/mL for cinnamon leaf oil (r^2^ = 0.9949) < 5.82 µg/mL for ascorbic acid (r^2^ = 0.9668) < 6.03 µg/mL for Trolox (r^2^ = 0.9925) < 6.86 µg/mL for BHA (r^2^ = 0.9957) < 7.70 µg/mL for α-tocopherol (r^2^ = 0.9961) < 49.50 µg/mL (r^2^ = 0.9810) for BHT. The low EC_50_ values reflect high and effective DPPH^•^ scavenging capabilities (Table 4 and Figure 3A).

As shown in Figure 3B, cinnamon (*C. zeylanicum*) leaf oil exhibited effective ABTS radical scavenging ability in a concentration-dependent manner (10–20 µg/mL, *p* < 0.001). The IC_50_ value of cinnamon leaf oil in the ABTS^•+^ removal assay was calculated as 12.53 µg/mL (r^2^ = 0.9827) (Table 4). When EC_50_ values were evaluated for standard molecules, the following sequence was observed: 6.35 µg/mL for BHA (r^2^ = 0.9746) < 11.74 µg/mL, for ascorbic acid (r^2^ = 0.9983) < 12.60 µg/mL for BHT (r^2^ = 0.9995) < 16.50 µg/mL for Trolox (r^2^ = 0.9775) < 18.72 µg/mL for α-tocopherol (r^2^ = 0.9347) (Figure 3B).

### 3.4. Inhibition of Enzymes by Cinnamon Leaf Oil

The inhibition results for the enzymes used are listed in Table 5. Cinnamon (*C. zeylanicum*) leaf oil inhibited AChE with an IC_50_ value of 16.03 µM (r^2^ = 0.9874). The tacrine standard inhibitor, used for comparison, inhibited AChE with an IC_50_ value of 7.58 µM (r^2^ = 0.9074). As seen in Table 4, cinnamon leaf oil had a moderate inhibitory effect on α-amylase, with an IC_50_ value of 553.07 µM (r^2^ = 0.9685). The IC_50_ value of cinnamon leaf oil against cytosolic and predominant hCA II isoenzyme was calculated as 25.41 ± 1.10 nM (Table 5). Acetazolamide (AZA), a clinical CA isoenzyme inhibitor used for comparison, inhibited cytosolic and predominant hCA II isoforms with an IC_50_ value of 4.41 ± 0.35 nM.

## 4. Discussion

Herbal extracts are commonly used in traditional and modern medicines owing to their beneficial health effects and healing properties, such as anti-aging, anti-fatigue, anti-cancer, and anti-hepatitis effects, as well as their ability to reduce blood serum lipid and glucose levels, and immunomodulation [83]. More than 25% of the drugs available worldwide are prepared from medicinal plants. Plants are rich in phenolic compounds, which are natural and active secondary plant metabolites. Recently, they have attracted great interest in pharmaceutical applications [84]. Currently, an increasing amount of scientific research is being conducted on the health benefits of naturally derived phenolics and flavonoids [36]. Consuming plants and foods rich in phenolic compounds has beneficial biological effects, including antioxidant, antimicrobial, and anti-inflammatory effects [85]. Numerous bioanalytical analyses are available for identifying and establishing a link between phenolic compounds and their applications. Due to its rich active phenolic content, the antioxidant activity of cinnamon leaf oil is expected to be high.

Owing to its high sensitivity, LC-HRMS is one of the most widely used chromatographic methods for the quantitative analysis of phenolic compounds in plant extracts [86]. Moreover, the optimization of various experimental parameters, such as the extraction solvent, column, mobile phase, and LC-HRMS conditions, is extremely important in the analysis of the phenolic content of oils obtained from plants [87]. The phenolic compounds are presumably responsible for the antioxidant ability of cinnamon leaf oil [88,89]. Phenolics extracted from plant materials have many effective biological functions, such as metal binding, ROS scavenging, reduction, and H-atom donation [90]. The most abundant phenolic compounds in plant oil were found to be isosakuranetin, hispidulin, and chlorogenic acid. Isosakuranetin is a 4′-O-methylated flavonoid, found in citrus fruits. It is consumed daily in the human diet as a constituent of a variety of plant components. Isosakuranetin has been previously described to have antimycobacterial, antioxidant, antifungal, antibacterial, anticancer, neuroprotective, and antiallergic properties [91]. Hispidulin (4′,5,7-trihydroxy-6-methoxyflavone) is an abundantly found flavonoid in medicinal plants, which has been widely used in medicine as an antioxidant [92]. Hispidulin was measured as the second dominant phenolic compound (6.61 mg/L oil) in cinnamon leaf oil. Chlorogenic acid, which is one of the abundant phenolic acids in plants, especially in coffee and tea, has been reported to have strong antioxidant activity as well as many pharmacological activities in many preclinical and clinical studies [93].

The reducing capacity of plant-derived oils contributes to their biological activities. Because of their high reducing capacity, these oils reduce oxidative stress and neutralize ROS [94]. The ferric ion (Fe^3+^) reduction method can be used to directly determine the reducing ability of plant-derived oils or extracts. The addition of cinnamon leaf oil to solutions containing Fe^3+^ ions under experimental conditions causes the formation of blue-colored Fe_4_[Fe(CN^−^)_6_]_3_, which can absorb light at 700 nm [95]. The color of the test mixture, along with the formation of this chromophore complex, reveals the reducing capability of the plant extracts in different color scales from yellow to green [96]. Cinnamon leaf oil demonstrated a strong and effective reducing capacity in Fe_4_[Fe(CN^−^)_6_]_3_, Fe^3+^-TPTZ, and Cu^2+^ reduction assays. The results demonstrate that cinnamon leaf oil has an effective Fe^3+^-reducing ability and that the e-donor property neutralizes the harmful effects of ROS and free radicals. This reductive potential was higher than that of BHT, Trolox, and α-tocopherol but close to that of BHA and ascorbic acid. The CUPRAC method, another reduction test used in our study, is a low-cost, selective, stable, and fast method for plant extracts and oils, independent of chemical components and hydrophobicity [97]. The final reduction test used in this study was the FRAP reduction method. As with the prior reduction assays, high absorbance values reflect the high reduction potential of the complex. In addition, the FRAP method should be applied in acidic media to maintain the solubility of Fe^3+^ ions [98,99].

The ABTS^•+^ and DPPH^•^ removal assays are the most useful and widely used radical removal assays. They are used to evaluate the radical scavenging capacity of materials in the food and pharmacology sectors. In a recent study, IC_50_ values of methanol and aqueous extracts of *M. pulegium* were calculated as 18.52 and 15.37 μg/mL, respectively [100]. The IC_50_ values for aqueous and ethanol extracts of *C. verum*, another cinnamon type, were determined to be 15.71 and 21.25 μg/mL, respectively [48]. In a prior study, IC_50_ values for methanol and aqueous extracts of *S. pilifera*, widely distributed in Anatolia, were 7.05 and 8.56 μg/mL, respectively [34]. The chromogenic ABTS radical scavenging assay can be easily applied to hydrophilic and lipophilic antioxidant compounds. The values for methanol and aqueous extracts of the *M. pulegium* plant were recorded as 7.92 and 9.37 μg/mL, respectively [100], and 3.52 and 4.76 μg/mL for methanol and aqueous extracts of *S. pilifera*, respectively [34]. In a recent study, the values were found to be 5.52 and 5.79 μg/mL for water and ethanol extracts of *C. verum*, respectively [48].

When the enzyme inhibition results were evaluated and compared with standard inhibitors, cinnamon leaf oil was found to have an effective inhibition capacity against AChE, α-amylase, and hCA II, which are associated with global metabolic diseases, such as AD and T2DM. Enzyme inhibition is an effective approach in the treatment of several diseases [101]. Based on these results, cinnamon leaf oil was a more effective and suitable inhibitor of AChE than tacrine. In a recent study by our group, methanol and water extracts of the *M. pulegium* had IC_50_ values of 40.76 and 53.31 μg/mL, respectively, for AChE [100]. In another study, water and ethanol extracts of *C. verum* had an inhibitory effect on AChE with Ki values of 221.33 μg/mL and 110.26 μg/mL, respectively [48]. Similarly, ethanolic (IC_50_: 23.01 μg/mL) and water extracts (IC_50_: 25.66 μg/mL) of *Rhus coriaria* have a highly effective inhibitory action on AChE [102]. The inhibition of α-amylase by natural products can effectively reduce blood glucose levels, particularly in PPGL and T2DM. Cinnamon leaf oil showed a greater inhibitory effect (IC_50_: 22.800 mM) than acarbose, a standard inhibitor [103]. In a recent study, ethanol and dichloromethane/methanol extracts of bark and leaf of authenticated Ceylon cinnamon (*C. zeylanicum*) were studied for α-amylase, α-glucosidase, and inhibition effects. It was reported that these extracts exhibited IC_50_ values of 26.62–36.09 and 214–215 and μg/mL against AChE and α-amylase, respectively [104]. The inhibitory effects on digestive enzymes, including α-amylase, can reduce blood glucose levels. Moreover, this inhibition may have a significant therapeutic effect on regulating hyperglycemia associated with diabetes.

Plants are rich in biologically active phenolic compounds. Phenolic compounds are slightly acidic and convert into water-soluble phenolate anions by losing at least one proton (H^+^) from their hydroxyl groups (-OH). Plants rich in phenolics effectively inhibit CA [105]. In addition, phenolic compounds found in plants can inhibit CA isoforms due to the coordination of the Zn^2+^ ion in the active sites to phenolic -OH, -COOH, and -OCH_3_ groups in their phenolic rings. This physiologically dominant isoform is found almost everywhere in cells and is associated with many diseases such as glaucoma, edema, and epilepsy [106].

## 5. Conclusions

Cinnamon leaf oil was evaluated using several in vitro bioanalytical assays for its antioxidant properties and inhibitory effects on metabolic enzymes, such as AChE, α-amylase, and CA II, which are associated with diabetes, Alzheimer’s disease, and glaucoma. In addition, the potential active ingredients present in cinnamon leaf oil were determined using LC-HR/MS, GC/MS, and GC-FID. The most abundant phenolic compounds in the plant oil were found to be isosakuranetin, hispidulin, and chlorogenic acid. Moreover, as a result of GC-FID analysis of cinnamon leaf oil, it was determined that 72.98% of the existing oil was E-Cinnamaldehyde. The results show that cinnamon leaf oil can be a rich and useful source of biologically important biomolecules. In addition, cinnamon oil is rich in natural phenolic compounds, such as *E*-cinnamaldehyde, benzyl benzoate, β-caryophyllene, and *trans*-cinnamylacetate. LC-HR/MS analysis showed that cinnamon leaf oil has high amounts of natural phenolic compounds, such as luteo-lin-7-glycoside, ellagic acid, and naringenin.

## Figures and Tables

**Table 1:** Chemical composition and validation parameters of cinnamon (*C. zeylanicum*) leaf oil (mg/L oil) obtained using LC-HRMS.

Phenolic Compounds	Molecular Formula	m/z	Ionization Mode	Linear Range	Linear Regression Equation	LOD/LOQ	R^2^	Recovery	Phenolics	U%
Ascorbic acid	C_6_H_8_O_6_	175.0248	Negative	0.5–10	y = 0.00347x − 0.00137	0.39/1.29	0.9988	96.20	4.33	3.94
Epigallocatechin	C_15_H_14_O_7_	307.0812	Positive	0.3–5	y = 0.00317x + 0.000443	0.17/0.57	0.9947	102.22	1.38	3.09
Chlorogenic acid	C_16_H_18_O_9_	353.0878	Negative	0.05–10	y = 0.00817x + 0.000163	0.02/0.06	0.9994	96.68	0.60	3.58
Fumaric acid	C_4_H_4_O_4_	115.0037	Negative	0.1–10	y = 0.00061x − 0.0000329	0.05/0.17	0.9991	97.13	4.39	2.88
Verbascoside	C_29_H_36_O_15_	623.1981	Negative	0.1–10	y = 0.00758x + 0.000563	0.03/0.1	0.9995	96.19	0.05	2.93
Orientin	C_21_H_20_O_11_	447.0933	Negative	0.1–10	y = 0.00757x + 0.000347	0.01/0.03	0.9993	96.22	0.31	3.67
Caffeic acid	C_9_H_8_O_4_	179.0350	Negative	0.3–10	y = 0.0304x + 0.00366	0.08/0.27	0.9993	94.51	0.46	3.74
Luteolin-7-rutinoside	C_27_H_30_O_15_	593.1512	Negative	0.1–10	y = 0.00879x + 0.000739	0.01/0.03	0.9988	93.05	-	3.06
Luteolin 7-glycoside	C_21_H_20_O_11_	447.0933	Negative	0.1–7	y = 0.0162x + 0.00226	0.01/0.03	0.9961	96.31	0.30	4.14
Rutin	C_27_H_30_O_16_	609.1461	Negative	0.05–10	y = 0.00329x − 0.00005576	0.01/0.03	0.999	96.97	0.26	3.07
Rosmarinic acid	C_18_H_16_O_8_	359.0772	Negative	0.05–10	y = 0.00717x − 0.0003067	0.01/0.03	0.9992	99.85	3.25	3.77
Hyperoside	C_21_H_20_O_12_	463.0882	Negative	0.05–10	y = 0.0072x − 0.00003096	0.01/0.03	0.9995	96.62	0.56	3.46
Apigenin 7-glycoside	C_21_H_20_O_10_	431.0984	Negative	0.3–7	y = 0.0246x + 0.00306	0.01/0.03	0.9962	96.07	0.01	2.86
Ellagic acid	C_14_H_6_O_8_	300.9990	Negative	0.05–10	y = 0.0085x − 0.000612	0.03/1	0.9994	101.49	0.27	3.59
Quercitrin	C_21_H_20_O_11_	447.0933	Negative	0.05–10	y = 0.0179 + 0.0003331	0.01/0.03	0.999	97.00	0.93	4.20
Quercetin	C_15_H_10_O_7_	301.0354	Negative	0.1–10	y = 0.0509x + 0.00467	0.01/0.03	0.9978	96.41	0.06	3.78
Herniarin	C_10_H_8_O_3_	177.0546	Positive	0.1–7	y = 0.309x + 0.0266	0.01/0.03	0.9983	92.92	0.03	2.95
Salicylic acid	C_7_H_6_O_3_	137.0244	Negative	0.3–10	y = 0.0361x + 0.00245	0.01/0.03	0.9982	92.88	7.82	3.89
Naringenin	C_15_H_12_O_5_	271.0612	Negative	0.1–10	y = 0.0281x + 0.00182	0.01/0.03	0.9995	86.65	0.38	1.89
Luteolin	C_15_H_10_O_6_	285.0405	Negative	0.1–10	y = 0.117x + 0.00848	0.01/0.03	0.9981	96.98	1.65	4.20
Apigenin	C_15_H_10_O_5_	269.0456	Negative	0.3–10	y = 0.104x + 0.0199	0.01/0.03	0.9998	81.55	0.40	3.42
Hispidulin	C_16_H_12_O_6_	301.0707	Positive	0.05–10	y = 0.02614x + 0.0003114	0.01/0.03	0.9993	98.36	6.61	2.87
Isosakuranetin	C_16_H_14_O_5_	285.0769	Negative	0.05–10	y = 0.0235x + 0.000561	0.01/0.03	0.9992	96.56	9.98	3.41
Penduletin	C_18_H_16_O_7_	343.0823	Negative	0.3–10	y = 0.0258x + 0.00253	0.01/0.03	0.9991	83.43	0.71	3.20
CAPE	C_17_H_16_O_4_	283.0976	Negative	0.3–7	y = 0.255x + 0.0477	0.01/0.03	0.9964	94.42	0.13	3.13
Chrysin	C_15_H_10_O_4_	253.0506	Negative	0.05–7	y = 0.0964x − 0.0002622	0.01/0.03	0.999	87.92	0.17	3.24
Quillaic acid	C_30_H_46_O_5_	485.3273	Negative	0.05–10	y = 0.00781x − 0.0001318	0.01/0.03	0.9992	90.29	1.47	2.56
Caryophyllene oxide	C_15_H_24_O	221.1900	Positive	3–7	y = 0.00151x + 0.00692		0.9909	96.87	1.53	4.05

**Table 2:** Chemical composition of the essential oil obtained from cinnamon (*C. zeylanicum*) leaf using GC-MS.

RT	Essential Oils	Contents (%)
937	*α*-Pinene	1.00
953	Camphene	0.34
986	*β*-Pinene	0.38
1008	Phellandrene	0.70
1026	*p*-Cymene	1.48
1097	Linalool	1.80
1193	*α*-Terpineol	0.48
1235	*Z*-Cinnamaldehyde	1.10
1281	Safrole	1.18
1284	*E*-Cinnamaldehyde	72.98
1365	Eugenol	1.48
1376	α-Copaene	0.77
1420	β-Caryophyllene	3.45
1433	*trans*-Cinnamylacetate	3.36
1458	*α*-Humulene	0.63
1525	Acetyleugenol	1.58
1586	(−)-Caryophyllene oxide	0.98
	Total	97.70

**Table 3:** Fe^3+^, Cu^2+^, and Fe^3+^-TPTZ reducing ability of cinnamon oil and standards at 50 μg/mL concentration (BHA: butylated hydroxyanisole, BHT: butylated hydroxytoluene).

Antioxidants	Fe^3+^ Reducing *		Cu^2+^ Reducing *		Fe^3+^-TPTZ Reducing *	
	λ_700_	r^2^	λ_450_	r^2^	λ_593_	r^2^
BHA	2.292 ± 0.012	0.9993	2.418 ± 0.018	0.9887	1.172 ± 0.014	0.9605
BHT	2.136 ± 0.090	0.9957	1.953 ± 0.045	0.9998	0.690 ± 0.008	0.9645
Trolox	1.514 ± 0.066	0.9963	1.800 ± 0.096	0.9974	1.180 ± 0.032	0.9732
α-Tocopherol	0.862 ± 0.038	0.9996	0.851 ± 0.046	0.9994	0.918 ± 0.011	0.9904
Ascorbic acid	2.298 ± 0.086	0.9659	0.983 ± 0.048	0.9822	1.257 ± 0.024	0.9869
Cinnamon leaf oil	2.190 ± 0.039	0.9741	1.918 ± 0.031	0.9992	1.900 ± 0.021	0.9725

* All values are averages of three parallel measurements (*n* = 3) and presented as mean ± SD.

**Table 4:** IC_50_ values (μg/mL) of 1,1-diphenyl-2-picryl-hydrazyl free radical (DPPH^•^) scavenging and 2,2′-azino-bis(3-ethylbenzthiazoline-6-sulfonic acid (ABTS^•+^) scavenging activities of cinnamon (*C. zeylanicum*) leaf oil and standards.

Antioxidants	DPPH^•^ Scavenging		ABTS^•+^ Scavenging	
	IC_50_	r^2^	IC_50_	r^2^
BHA	6.86	0.9949	6.35	0.9746
BHT	49.50	0.9957	12.60	0.9995
Trolox	6.03	0.9925	16.50	0.9775
α-Tocopherol	7.70	0.9961	18.72	0.9347
Ascorbic acid	5.82	0.9668	11.74	0.9983
Cinnamon leaf oil	4.78	0.9344	5.21	0.9563

**Table 5:** The half maximal inhibition concentration (IC_50_; µg/mL) values of cinnamon (*C. zeylanicum*) leaf oil against acetylcholinesterase, α-amylase, and carbonic anhydrase II enzymes.

Enzymes	Cinnamon Leaf Oil		Standard Inhibitors	
	IC_50_	r^2^	IC_50_	r^2^
α-Amylase ^1^	553.07	0.9058	7.54	0.9074
Acetylcholinesterase ^2^	16.03	0.9874	8.82	0.9836
Carbonic anhydrase II ^3^	243.24	0.9092	9.96	0.9930

^1^ Acarbose was used as a positive control for α-glycosidase and α-amylase enzymes. ^2^ Tacrine was used as a positive control for acetylcholinesterase and butyrylcholinesterase enzymes. ^3^ Acetazolamide (AZA) was used as a positive control for carbonic anhydrase II isoenzyme.

## Data Availability

Data are available in a publicly accessible repository.
